# Risk of adverse newborn outcomes among women who experienced physical and psychological intimate partner abuse during pregnancy in Ghana's northern region

**DOI:** 10.1016/j.heliyon.2023.e15391

**Published:** 2023-04-10

**Authors:** Michael Boah, Nashiru Abdulai, Abdul-Nasir Issah, Daudi Yeboah, Mary Rachael Kpordoxah, Jevaise Aballo, Martin Nyaaba Adokiya

**Affiliations:** a Department of Epidemiology, Biostatistics, and Disease Control, School of Public Health, University for Development Studies, Tamale, Ghana; b Department of Global and International Health, School of Public Health, University for Development Studies, Tamale, Ghana; c Nanton District Assembly, P.O. Box 1, Tamale, Ghana; d Department of Health Services, Policy, Planning, Management, and Economics, School of Public Health, University for Development Studies, Tamale, Ghana; e United Nations Children Fund (UNICEF). Ghana Country Office, P.O. Box AN 5051, Accra, Ghana

**Keywords:** Intimate partner violence, Newborn outcomes, Pregnant women, Ghana

## Abstract

**Background:**

Intimate partner violence (IPV) is common worldwide. However, the health effects of exposure to IPV during pregnancy are significantly more severe. We investigated the relationship between exposure to IPV during pregnancy and the risk of preterm and low birthweight births among women in Ghana's northern region.

**Methods:**

We recruited 402 postnatal women aged 15–49 years from five selected public health facilities in the Tamale Metropolis of the northern region of Ghana. Using Kobo Collect, information on a wide range of factors, including exposure to IPV during the last pregnancy and pregnancy outcomes, was collected electronically. Multiple logistic regression analyses were conducted in Stata to determine the associations between prenatal exposure to IPV and binary measures of gestational age at birth and birthweight.

**Results:**

Overall, 35.1% (95% CI: 30.5, 39.9) of the respondents experienced IPV during their recent pregnancy; 6.7% (95% CI: 4.6, 9.6) experienced physical IPV; and 34.8% (95% CI: 30.3, 39.6) experienced psychological IPV. The prevalence of preterm and low birthweight deliveries was 18.9% (95% CI: 15.4, 23.1) and 9.0% (95% CI: 6.5, 12.2), respectively. Prenatal exposure to IPV was linked to poor newborn outcomes by multivariable binary regression models. Women who suffered IPV during their last pregnancy were three times more likely to deliver low birthweight babies (AOR = 3.12: 95% CI: 1.42, 6.84). Exposed women were also about twice as likely to deliver prematurely, although this association was not statistically significant (AOR = 1.81; 95% CI: 0.97, 3.38).

**Conclusion:**

Exposure to IPV during pregnancy increases a woman's risk of delivering prematurely and having a low birthweight baby. IPV screening should be a regular part of ANC, so that pregnant women who are experiencing IPV can be monitored and supported to avoid adverse outcomes for their babies.

## 1 Introduction

Physical, emotional, and sexual violence against women is a major violation of human rights and a global public health issue of concern. Intimate Partner Violence (IPV) is defined as “behaviour within an intimate relationship that causes physical, sexual, or psychological harm, including acts of physical aggression, sexual coercion, psychological abuse, and controlling behaviours” [1]. While we recognise that women can also perpetrate violence against their partners and that IPV occurs in same-sex relationships, existing evidence indicates that men are more likely than women to perpetrate IPV [2]. It is estimated that 27% of ever-partnered women aged 15 or older have experienced IPV at some point in their lives [3]. There is also evidence of regional variations in the prevalence of IPV. A systematic review and meta-analysis of cross-sectional studies in sub-Saharan Africa (SSA) has reported that 44% of women in the region have experienced IPV at some time in their lives, with emotional violence being the highest (29.4%), followed by physical violence (25.9%), and the lowest being sexual violence (18.8%) [4]. Unfortunately, most people in the region, including some women, accept violence against women. A multi-country study reported that about 29% of women in SSA endorse the beating of women under certain circumstances, ranging from nearly 17% in Malawi to as high as 82.3% in Mali. The same study reported a rate of 30% for Ghana [5].

Although IPV can occur at any point in a woman's life, it is of particular concern during pregnancy due to the negative effects it can have on both the mother and her unborn child. The prevalence of IPV during pregnancy varies by country, but international comparisons have proven difficult due to varying study methodologies and definitions. Reviews, however, established that IPV during pregnancy ranged from 2 to 57% among African countries [6], 3–44% among Latin American and Caribbean countries [7], and 6–33% among Asian countries [8,9]. These statistics demonstrate that IPV during pregnancy is widespread. IPV during pregnancy is claimed to be influenced by numerous factors, including but not limited to unintended pregnancies, socioeconomic status, women's decision-making power, alcohol and drug use, pregnancy-related depression, the type of marriage, the partner's level of education, and acceptance of violence [[6], [7], [8],^10^].

Studies have demonstrated the multifaceted effects of violence on pregnant women. Women who experience IPV during pregnancy have more symptoms of depression and anxiety and overall poorer psychosocial health than those who do not [[11], [12], [13], [14], [15]]. In addition, pregnant women who are exposed have an increased risk of arterial hypertension, premature rupture of membranes, preeclampsia, intrauterine growth restriction (IUGR), placental abruption, risky sexual behaviours, and genitourinary tract infections [[16], [17], [18], [19]]. Women who experienced prenatal IPV were less likely to use antenatal care (ANC) services, or if they did, they made fewer contacts and were less likely to take iron and folic acid before delivery than if they did not experience IPV during pregnancy [[20], [21], [22], [23]].

Regarding IPV during pregnancy and the risk of adverse newborn outcomes, specifically preterm birth and having a low birthweight baby, the evidence is inconclusive. Several epidemiological studies have linked exposure to IPV during pregnancy to an increased risk of preterm birth and low birthweight [18,[24], [25], [26], [27], [28], [29]]. Other studies have found no significant association between maternal exposure to IPV during pregnancy and these outcomes [[30], [31], [32]]. Importantly, according to the literature, preterm delivery and low birthweight, are major causes of newborn morbidity and mortality in children under five years of age and contribute to various complications later in life [[33], [34], [35]].

Men's tolerance of IPV against women varies by region in Ghana. According to a recent study, men's justification for IPV in the form of wife-beating has decreased in the country from 32% in 2003 to 12.4% in 2014, with the northern region reporting the second highest rate of 30% after the upper east region in 2014 [36]. The study also found that northern men were significantly more likely than their southern counterparts to justify domestic violence. In addition to its tolerance for domestic abuse, the northern region is known for having a relatively high rate of poor neonatal outcomes. According to the literature, low birth weight, for example, is 30% in the northern region [37] compared to 14% in the upper east [38], 8.2% in the upper west [39] and 10–23% in the regions in the southern and coastal areas of the country [[40], [41], [42]]. However, it is not known if IPV during pregnancy is linked to adverse outcomes for babies in the region.

This study examined the association between prenatal exposure to IPV and the risk of adverse newborn outcomes, such as preterm birth and having a low birthweight baby, in the Tamale Metropolitan Area of northern Ghana.

## 2 Material and methods

### 2.1 Study setting and design

**Fig. 1:**
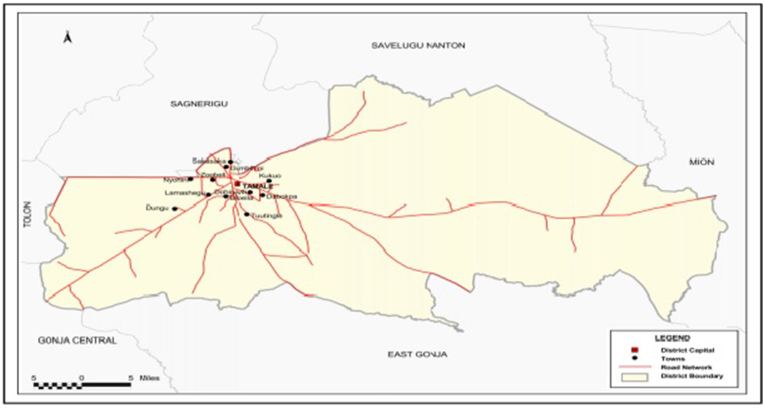
Map of Tamale Metropolis. Source: Ghana statistical service, 2014.

A health facility-based retrospective cross-sectional study was conducted in five selected public health facilities in the Tamale Metropolis in the northern region of Ghana. The Tamale Metropolis is one of the sixteen districts in the northern region, with Tamale as the capital. It shares boundaries with Savelugu Municipality to the North, East Gonja Municipal to the South, Central Gonja District to the South-West, Yendi Municipal Assembly to the East, and Tolon District to the West (Fig. 1) [43]. In terms of public health infrastructure, the Metropolis has three hospitals, including one teaching hospital, eight health centres, and eighteen Community-based Health Planning and Services (CHPS) serving a population of 374,744. More than 80% of the population in the Metropolis lives in urban areas.

In general, the northern region of Ghana, where the current study's setting is located, is one of the most disadvantaged regions in the country. According to national statistics [44], the northern region has the highest rate of illiteracy among both men and women, and more than half of the population in the region is in the lowest wealth quintile. It is also the region with the highest rate of polygynous unions in the country.

### 2.2 Sample

The study population was comprised of women who had delivered in the past 6–12 months prior to the study. This population was selected to minimise recall bias. In addition, according to Ghana's child welfare schedule, this is the time when mothers or caregivers actively bring their children to health facilities for growth monitoring and immunizations, which would allow the research team easy access to the participants. The sample size was determined using the formula:

(1) $$n=N/\left(1+Ne^{2}\right)$$

where: ‘n’ is the sample size required for the study; ‘N’ is the estimated cumulative number of deliveries (4,292) for the facilities selected; and ‘e’ is the acceptable margin of error (5%). A total of 402 women were needed, which included a 10% non-response rate. The study included only partnered women who gave birth to single babies at the facility and had babies aged 6–12 months who were receiving postnatal or child welfare care services, had complete health records, and were willing to participate.

### 2.3 Selection of facilities and respondents

Multiple sampling methods were used to select the public health facilities and the respondents in this study. Two secondary public health facilities (i.e., Tamale Central and Tamale West hospitals) were purposefully selected because they provided ANC and delivery services to a significant proportion of reproductive women in the area. In addition to the two secondary-level facilities, three health centres (Datoyili Health Center, Bilpiela Health Center, and Vittin Reproductive and Child Health) were randomly selected from the eight in the Metropolis. The sample for the study was distributed to the facilities using the proportion-to-size technique.

Using a systematic random sampling technique, the respondents were then chosen. The postnatal register was used to compile a list of partnered women who had given birth in the 6–12 months prior to the survey at each of the chosen health facilities. Then a sampling interval was calculated using the sampling frame by dividing the total number of eligible women by the sample size required for the facility. As a starting point, a participant was chosen at random from the first ten names in the sampling frame. The sampling interval was then used to choose respondents until the desired sample size was reached.

### 2.4 Data collection and tool

The data were collected electronically using the Kobo Collect application installed on Android-powered mobile phones. The data collection tool was pretested among 40 women (10% of the total sample size) from a health facility outside the study setting. The pretesting ensured the validity of the questionnaire in gathering the required information. All ambiguous questions were revised. The questionnaire collected data on sociodemographic and economic characteristics, obstetric history, and other maternal health-related factors. Data on the woman's gestation at delivery, haemoglobin levels, and birthweight of the child at delivery were collected by reviewing the ANC record book. Trained research assistants with a degree in nursing collected the data under the supervision of the research team. The data collection took place in June 2022.

### 2.5 Measures

#### 2.5.1 Assessing exposure to intimate partner violence during pregnancy (main exposure variable)

To measure exposure to violence, the respondents were asked a series of questions on violent acts that occurred during their recent pregnancies. The questions were adopted from the domestic violence module questionnaire used by the Demographic and Health Survey (DHS) program (https://dhsprogram.com/pubs/pdf/DHSQMP/domestic_violence_module.pdf.pdf). Information on experience of any physical violence during pregnancy was collected by asking women “whether her partner ever pushed, shook or had something thrown at her; slapped her; punched her with his fist or with something that could hurt her, kicked her; dragged her or beat her up; tried to choke her or burn her on purpose; threatened or attacked her with a knife, gun, or any other weapon; or twisted her arm or pulled her hair.” Information on psychological IPV was gathered from asking women “whether her partner ever humiliated, threatened with harm, or insulted or made to feel bad” during her most recent pregnancy. The response options were no and yes. We created dichotomous variables for the experience of physical and psychological IPV. The experience of any physical or psychological violence (coded as 1) was considered if a woman reported at least one act of violence from her partner during the index pregnancy. Women who reported never experiencing any of these incidents during pregnancy were not considered to have been exposed to physical or psychological violence (coded as 0).

### 2.6 Outcome variable

The dependent variables in the study were the gestation at birth and the birthweight of the baby. Specifically, the two adverse newborn outcomes examined were preterm birth and having a low birthweight baby. Information on these outcomes was retrieved from the ANC card of the mother. These variables were dichotomized to create preterm and low birthweight outcomes. Gestation at birth was coded as “1” (preterm) if the gestation at birth was below 37 weeks and coded as “0” if it was ≥37 weeks. Similarly, birth weight was coded as “1” if it was less than 2.5 kg (or 2500 g) and coded as “0” if it was ≥2.5 kg. The categorization of the variables was guided by the existing WHO literature [45].

### 2.7 Covariates

The analysis was adjusted for several potential confounding factors. These include women's sociodemographic and economic factors, obstetrical factors, and health-related behavioural factors. The sociodemographic and economic factors included women's current age (in years) and education. Obstetric factors included the number of pregnancies and parity. The gestation at ANC initiation, the number of ANC contacts before delivery, the experience of complications with the previous pregnancy (yes/no), the experience of any malaria episode during the previous pregnancy (yes/no), and anaemia status in the first and second trimesters (yes/no) were all health-related behavioural factors considered in the current study.

### 2.8 Data analysis

We first performed descriptive statistics on the background characteristics of the respondents and the IPV variables to examine the distribution of these variables. Then univariate analyses were performed using a Chi-square test or Fishers' test as appropriate to examine the association between the IPV variables and preterm birth and low birthweight. Lastly, we performed binary multiple logistic regression analyses to evaluate the association between women's experience of IPV during pregnancy and the risk of preterm and low birthweight births. We entered all the covariates into the multiple logistic regression models to adjust for potential confounding by these factors. The adjusted odds ratio (AOR) was reported with a 95% confidence interval (CI). Statistical significance was pegged at a probability value of less than 0.05. The reported P-values were two-tailed. All the statistical analyses were performed using Stata/IC 15.0 for Windows (StataCorp LLC, College Station, Texas, USA).

## 3 Results

### 3.1 Background characteristics of the respondents in the current study

The background characteristics of the respondents in the study, including sociodemographic and economic characteristics, as well as obstetric and other health-related characteristics, are presented in Table 1. The mean age was 31.3 (±5.1 years), ranging from 19 to 49 years. More than half (58.5%) of the respondents were in the age group of 25–34 years, 64.2% of them received no formal education, and 95.8% were affiliated with Islam. According to the results on obstetric and health-related characteristics, 17.2% had at least 5 pregnancies, and 21.6% had at least 4 living children. In addition, 11.9% of the respondents made their first ANC contact in the first trimester, and 37.6% made at least eight contacts before delivery. The results also showed that 20.4% and 32.1% of the women were anaemic in the first and second trimesters, respectively (Table 1).

**Table 1:** Background characteristics of the respondents in the current study (N = 402).

Variable	Frequency	Percentage
Age group (years) mean 31.3 (±5.1 years)		
15–24	47	11.7
25–34	235	58.5
35–49	120	29.9
Education		
No formal education	258	64.2
Some formal education	144	35.8
Religious affiliation		
Christian	17	4.2
Islam	385	95.8
Ethnicity		
Dagomba	343	85.3
Gonja	34	8.5
Mamprusi	10	2.5
Others^a^	15	3.7
Employment		
Unemployed	96	23.9
Employed	306	76.1
Number of pregnancies		
1	88	21.9
2	99	24.6
3	85	21.1
4	61	15.2
≥5	69	17.2
Parity		
1	111	27.6
2	127	31.6
3	77	19.2
≥4	87	21.6
Gestation at first ANC contact		
1st trimester	48	11.9
2nd trimester	339	84.3
3rd trimester	15	3.7
Number of ANC contacts before delivery		
1–3	20	5.0
4–7	231	57.5
8+	151	37.6
Experienced complications with last pregnancy		
No	189	47.0
Yes	213	53.0
Had malaria episode during recent pregnancy		
No	285	70.9
Yes	117	29.1
Anaemia at 1st trimester		
No	320	79.6
Yes	82	20.4
Anaemia at 2nd trimester		
No	273	67.9
Yes	129	32.1

a Others: Frafra, Fulani, Akan, Wala, Hausa, and Dagaaba

### 3.2 Prevalence of physical and psychological intimate partner violence during pregnancy

Table 2 summarises the prevalence of each abuse variable. The results show that 6.7% (95% CI: 4.6, 9.6) of the women experienced acts of physical IPV and 34.5% (95% CI: 30.3, 39.6) experienced acts of psychological IPV during the last pregnancy. Overall, 35.1% (95% CI: 30.5, 39.9) of the sample experienced at least one form of IPV during pregnancy; 28.6% experienced only one form of IPV; and 6.5% experienced the two forms of IPV examined. The distribution of the responses used to construct these IPV variables is presented as supplementary material (see Supplemental Material Table 1).

**Table 2:** Prevalence of physical and psychological intimate partner violence during pregnancy (N = 402).

Scores for the number of acts of physical intimate partner violence during recent pregnancy	Frequency	Percentage
0	375	93.3
1	20	5.0
2	5	1.2
3	2	0.5
Exposure to any act of physical intimate partner violence		
No	375	93.3
Yes	27	6.7
Scores for the number of acts of psychological intimate partner violence		
0	262	65.5
1	127	31.4
2	11	2.7
3	2	0.5
Exposure to any act of psychological intimate partner violence		
No	262	65.5
Yes	140	34.5
Number of forms of IPV experienced during the most recent pregnancy		
0	261	64.9
1	115	28.6
2	26	6.5
Experience of any form of IPV during most recent pregnancy		
No	261	64.9
Yes	141	35.1

### 3.3 Prevalence of preterm delivery and low birthweight in the study

**Fig. 2:**
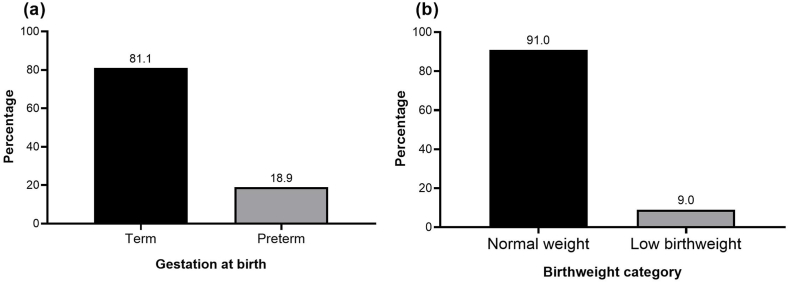
Prevalence of preterm delivery and low birthweight in the current study (N = 402).

The findings revealed that of the 402 women studied, 76 had preterm births and 36 had low birthweight babies, giving a prevalence of 18.9% (95% CI: 15.4, 23.1) for preterm delivery (Fig. 2a) and 9.0% (95% CI: 6.5, 12.2) for low birthweight (Fig. 2b). Further analysis revealed that 17.9% (95% CI: 14.4, 22.0) of the women had only one adverse pregnancy outcome, while 5.0% (95% CI: 3.2, 7.6) had the two adverse neonatal outcomes.

### 3.4 Distribution of preterm delivery by women's experience of intimate partner violence during their last pregnancy and other background characteristics

Women's exposure to IPV during pregnancy was associated with varying rates of preterm birth. A higher percentage of preterm births was recorded among women who suffered physical (59.3%), psychological (24.3%), and at least one form of IPV (24.8%) during their most recent pregnancy than those who did not (Table 3). Furthermore, the rate of preterm delivery also differed considerably by the women's age, number of pregnancies, parity, number of ANC contacts prior to delivery, a history of complications, and a malaria episode during the most recent pregnancy. Higher rates of preterm births were recorded among women in the age group of 15–24 (42.6%), women with one pregnancy (53.4%), and women who made 1–3 ANC visits during their most recent pregnancy (50.0%). In addition, preterm birth rates were higher among women who did not experience complications with their last pregnancy (31.2%) and those who did not experience any malaria episodes during their most recent pregnancy (12.0%).

**Table 3:** Distribution of preterm delivery by women's experience of intimate partner violence during their most recent pregnancy and other background characteristics (N = 402).

Variable	Preterm birth		P-value
	No	Yes	
Experienced physical IPV during pregnancy			<0.001
No	315 (84.0)	60 (16.0)	
Yes	11 (40.7)	16 (59.3)	
Experienced psychological IPV during pregnancy			0.044
No	220 (84.0)	42 (16.0)	
Yes	106 (75.7)	34 (24.3)	
Exposure to any form of IPV during recent pregnancy			0.026
No	220 (84.3)	41 (15.7)	
Yes	106 (75.2)	35 (24.8)	
Age group			<0.001
15–24	27 (57.4)	20 (42.6)	
25–34	184 (78.3)	51 (21.7)	
35–49	115 (95.8)	5 (4.2)	
Education			0.461
No formal education	212 (82.2)	46 (17.8)	
Some formal education	114 (79.2)	30 (20.8)	
Number of pregnancies			<0.001^b^
1	41 (46.6)	47 (53.4)	
2	86 (86.9)	13 (13.1)	
3	82 (96.5)	3 (3.5)	
4	53 (86.9)	8 (13.1)	
≥5	64 (92.8)	5 (7.2)	
Parity			<0.001^b^
1	61 (55.0)	50 (45.0)	
2	112 (88.2)	15 (11.8)	
3	71 (92.2)	6 (7.8)	
≥4	82 (94.3)	5 (5.7)	
Gestation at first ANC contact			0.100
1st trimester	34 (70.8)	14 (29.2)	
2nd trimester	281 (82.9)	58 (17.1)	
3rd trimester	11 (73.3)	4 (26.7)	
Number of ANC contacts before delivery			<0.001
1–3	10 (50.0)	10 (50.0)	
4–7	178 (77.1)	53 (22.9)	
8+	138 (91.4)	13 (8.6)	
Experienced complications with last pregnancy			<0.001
No	130 (68.8)	59 (31.2)	
Yes	196 (92.0)	17 (8.0)	
Had malaria episode during recent pregnancy			0.023
No	223 (78.2)	62 (21.8)	
Yes	103 (88.0)	14 (12.0)	
Anaemia at 1st trimester			0.155
No	264 (82.5)	56 (17.5)	
Yes	62 (75.6)	20 (24.4)	
Anaemia at 2nd trimester			0.660
No	223 (81.7)	50 (18.3)	
Yes	103 (79.8)	26 (20.2)	

^b^Fisher's test.

### 3.5 Distribution of low birthweight by women's experience of intimate partner violence during their most recent pregnancy and other background characteristics

There was a statistically significant association between women's exposure to IPV during pregnancy and the rate of low birthweight. The results showed that the rate of low birthweight was higher among women with a history of physical IPV (33.3%), psychological IPV (15.0%), or at least one form of IPV (14.9%). The rate of low birthweight delivery was higher among women in the age group of 15–24 (19.1%), women with one pregnancy (19.3%) and women of parity one (19.8%). Furthermore, a significant percentage of women who made 1–3 ANC contacts before delivery (20.0%) and women who were anaemic during the first trimester (15.9%) gave birth to low birthweight babies compared to their counterparts (Table 4).

**Table 4:** Distribution of low birthweight by women's experience of intimate partner violence during their most recent pregnancy and other background characteristics (N = 402).

Variable	Low birthweight		P-value
	No	Yes	
Experienced physical IPV during recent pregnancy			<0.001
No	348 (92.8)	27 (7.2)	
Yes	18 (66.7)	9 (33.3)	
Experienced psychological IPV during recent pregnancy			0.002
No	247 (94.3)	15 (5.7)	
Yes	119 (85.0)	21 (15.0)	
Exposure to any form of IPV during recent pregnancy			0.002
No	246 (94.3)	15 (5.7)	
Yes	120 (85.1)	21 (14.9)	
Age group (years)			0.034
15–24	38 (80.9)	9 (19.1)	
25–34	217 (92.3)	18 (7.7)	
35–49	111 (92.5)	9 (7.5)	
Education			0.744
No formal education	234 (90.7)	24 (9.3)	
Some formal education	132 (91.7)	12 (8.3)	
Number of pregnancies			0.006^b^
1	71 (80.7)	17 (19.3)	
2	94 (94.9)	5 (5.1)	
3	81 (95.3)	4 (4.7)	
4	55 (90.2)	6 (9.8)	
≥5	65 (94.2)	4 (5.8)	
Parity			<0.001^b^
1	89 (80.2)	22 (19.8)	
2	122 (96.1)	5 (3.9)	
3	72 (93.5)	5 (6.5)	
≥4	83 (95.4)	4 (4.6)	
Gestation at first ANC contact			0.633
1st trimester	42 (87.5)	6 (12.5)	
2nd trimester	310 (91.4)	29 (8.6)	
3rd trimester	14 (93.3)	1 (6.7)	
Number of ANC contacts before delivery			0.019^b^
1–3	16 (80.0)	4 (20.0)	
4–7	206 (89.2)	25 (10.8)	
8+	144 (95.4)	7 (4.6)	
Experienced complications with last pregnancy			0.076
No	167 (88.4)	22 (11.6)	
Yes	199 (93.4)	14 (6.6)	
Had malaria episode during recent pregnancy			0.570
No	258 (90.5)	27 (9.5)	
Yes	108 (92.3)	9 (7.7)	
Anaemia at 1st trimester			0.014
No	297 (92.8)	23 (7.2)	
Yes	69 (84.1)	13 (15.9)	
Anaemia at 2nd trimester			0.836
No	248 (90.8)	25 (9.2)	
Yes	118 (91.5)	11 (8.5)	

^b^Fisher's test.

### 3.6 Association between exposure to intimate partner violence during pregnancy and the risk of preterm birth and having a low birthweight baby

Table 5 shows the results of a binary logistic regression analysis examining the association between exposure to IPV during pregnancy and the risk of preterm and low birthweight births. After controlling for demographic, economic, and obstetric characteristics, the results indicated that women's exposure to IPV during pregnancy was related to the probability of delivering preterm and having a low birthweight baby. However, only the latter result was statistically significant at the alpha level of 5%. Women who experienced intimate partner violence during their most recent pregnancy had a higher chance of delivering preterm (AOR = 1.81; 95% CI: 0.97, 3.38) and low birthweight babies (AOR = 3.12: 95% CI: 1.42, 6.84). We found that, among the covariates, the number of ANC contacts prior to delivery was linked with the likelihood of both preterm and low birthweight births. The results demonstrated that the risk of preterm and low birthweight births decreases as the number of contacts increases. Mothers with at least eight contacts, for example, were 95% less likely to deliver preterm (AOR = 0.05; 95% CI: 0.01, 0.20) and 79% less likely to deliver a baby with a low birthweight (AOR = 0.21; 95% CI: 0.05, 0.96).

**Table 5:** Association between exposure to IPV during pregnancy and the risk of preterm and low birthweight delivery among women (N = 402).

Variable	Preterm delivery		Low birthweight	
	AOR (95% CI)	P-value	AOR (95% CI)	P-value
Exposure to any form of IPV				
No	1.00		1.00	
Yes	1.81 (0.97, 3.38)	0.064	3.12 (1.42, 6.84)	0.005
Age group (years)				
15–24	1.00		1.00	
25–34	0.61 (0.27, 1.35)	0.223	0.42 (0.15, 1.18)	0.100
35–49	0.29 (0.07, 1.17)	0.082	0.98 (0.22, 4.32)	0.976
Education				
No formal education	1.00		1.00	
Some formal education	0.84 (0.43, 1.64)	0.610	0.75 (0.32, 1.76)	0.506
Number of pregnancies	0.80 (0.45, 1.40)	0.432	1.71 (0.93, 3.13)	0.082
Parity	0.61 (0.30, 1.24)	0.171	0.32 (0.15, 0.67)	0.003
Gestation at first ANC contact				
1st trimester	1.59 (0.32, 7.79)	0.570	2.43 (0.23, 25.10)	0.457
2nd trimester	0.40 (0.09, 1.70)	0.215	1.49 (0.17, 13.12)	0.717
3rd trimester	1.00		1.00	
Number of ANC contacts before delivery				
1–3	1.00		1.00	
4–7	0.21 (0.07, 0.70)	0.010	0.54 (0.14, 2.15)	0.383
8+	0.05 (0.01, 0.20)	<0.001	0.21 (0.05, 0.96)	0.045
Experienced complications with recent pregnancy				
No	1.00		1.00	
Yes	0.33 (0.15, 0.72)	0.005	0.51 (0.19, 1.38)	0.186
Malaria during recent pregnancy				
No	1.00		1.00	
Yes	0.97 (0.42, 2.22)	0.939	1.01 (0.38, 2.69)	0.978
Anaemia at 1st trimester				
No	1.00		1.00	
Yes	1.90 (0.91, 4.00)	0.088	3.27 (1.39, 7.70)	0.007
Anaemia at 2nd trimester				
No	1.00		1.00	
Yes	1.01 (0.52, 1.96)	0.981	1.26 (0.54, 2.98)	0.594

## 4 Discussion

The purpose of the study was to examine the link between exposure to abuse by an intimate partner during pregnancy and the risk of adverse neonatal outcomes in a cross-section of reproductive women in Ghana's northern region. From our study, more than one in three women suffered psychological violence and about 7% suffered physical IPV during their last pregnancy. In total, more than one-third of the women suffered some form of IPV during their last pregnancy. The rate of IPV among pregnant women in this study is within the range of 2–57% among African countries [6], 3–44% in the Latin American and Caribbean region [7], and 6–33% among Asian countries [8,9]. The results of the current study confirm that IPV is a common occurrence during pregnancy, affecting many women. Also, from a review and meta-analysis study, more women in SSA suffer psychological abuse than any other form of abuse [4]. The current study found that psychological IPV is highly prevalent compared to physical IPV in the study setting. We acknowledge that comparing rates of IPV across countries has been challenging due, in part, to varying methodologies, violence measures, and context. A community-based study in Ethiopia, for instance, examined IPV during pregnancy based on exposure to physical, psychological, and sexual abuse and reported a rate of 44.5% [46]. Using the three forms of IPV, a facility-based study in Kenya revealed a rate of 37%, which is somewhat lower than the percentage observed in Ethiopia [10]. Despite the obvious limitations of comparison, violence against women by an intimate partner is a violation of human rights, and the health repercussions are more severe for pregnant women because of their vulnerability and the effects on both mother and unborn baby [7,16,17,47,48].

In this study, about 19% of babies were born prematurely, and 9% had low birthweight, which is comparable to what has been documented in other African and non-African countries as well as around the globe [[49], [50], [51]]. The key finding of our study is that exposure to physical and/or psychological abuse during pregnancy is associated with the risk of adverse newborn outcomes. We found that women who experienced prenatal IPV had a twofold increased risk of delivering prematurely and a threefold increased risk of delivering a baby with a low birthweight compared to those who did not experience any form of violence, although the evidence for the former was statistically insignificant. This finding on the association between exposure to IPV during pregnancy and the risk of adverse newborn outcomes is consistent with some studies [16,24,28,47,51], but not with others [[30], [31], [32]].

There are some viable scientific and psychosocial explanations for the link between prenatal exposure to IPV and foetal outcomes [52]. Physical or psychological IPV can influence neonatal outcomes through physiological reactions to violence-related stress by releasing prostaglandin, which can cause premature contractions and delivery, or vasoconstrictors or cortisol, which can result in IUGR [7,53,54]. Additionally, high levels of cortisol are necessary to trigger the physiological processes involved in parturition and prepare the uterus’ myometrium for the effects of oxytocin [55]. Stress, on the other hand, triggers the release of cortisol before term, which could result in preterm labour and delivery. Placental abruption and premature rupture of membranes are among some of the pregnancy complications caused by direct physical assault and are linked to poor newborn outcomes [19,48,56]. Violence-related stress during pregnancy raises the levels of norepinephrine and cytokines, which cause the uterine and placental blood vessels to narrow and stay that way. This long-term vasoconstriction makes it harder for oxygen and nutrients to get to the foetus, which slows foetal growth [54].

Other explanations for the association between prenatal IPV and premature birth and low birthweight are that abused women suffer from high stress and poor mental health, which can lead to poor nutrition patterns and their consequences, such as anaemia, underweight, and poor gestational weight gain, potentially resulting in IUGR and low birthweight [7,27,[57], [58], [59]]. Poor diet due to psychological stress can increase underlying illnesses like hypertension and gestational diabetes or lead to pregnancy disorders like preeclampsia and eclampsia, which are risk factors for premature labour and low birthweight [7,19,60]. Also, women who suffer IPV typically lack decision-making power over their own health and are more likely to not register, to commence ANC late, and to underutilize ANC services, decreasing their chances of obtaining health interventions that promote better maternal and newborn health prior to birth [21,49,51,61].

Our study also identified additional significant predictors of preterm and low birthweight births, including parity, number of ANC contacts, history of prenatal problems, and anaemia during the first trimester of pregnancy, which either support or refute other research. Higher parity has been linked to an increased risk of low birthweight in northern and southern Ghana [37,42]. The relationships between fewer ANC contacts, low haemoglobin levels, and an increased risk of bad newborn outcomes are also consistent with findings from other studies [42,51,59,62,63]. In contrast, the result that women who had problems during their previous pregnancy were less likely to deliver prematurely contrasts with what has been reported in Ethiopia, where women who had complications had an increased risk of premature birth [49].

### 4.1 Study limitations

Our study yields significant findings that can inform policy decisions. However, the results must be evaluated in light of a number of limitations. There are various types of IPV, including sexual violence and controlling behaviours. The current study, however, only looked at psychological and physical IPV. Furthermore, the current study did not take into account several adverse pregnancy outcomes, such as stillbirths, intrauterine foetal death, and antepartum haemorrhage (APH). These outcomes should be included in future research in the study setting. A cross-section of reproductive women who were in a union (married or cohabiting) at the time of the study were recruited from public health institutions in the northern area of Ghana for the current study. Consequently, our findings are restricted to this particular sample of women. In addition, the cross-sectional methodology restricts the conclusions to associations, not causal links. IPV is a sensitive subject in the majority of circumstances. We therefore believe that some women may have underreported their exposure. We also feel that the retrospective collection of this information was susceptible to recollection bias. Despite this, the short recollection interval may have reduced the magnitude of bias. Last but not least, there are numerous known factors that can potentially affect birth outcomes, such as heat and air pollution exposure, pregnancy interval, body mass index, human immunodeficiency virus infection, and history of abortion among others [[64], [65], [66], [67]]. However, in the present study, we were unable to take into account all of these variables.

## 5 Conclusions

According to our study, more than one-third of pregnant women in the study setting suffered physical and/or psychological abuse from their intimate partner during their most recent pregnancy. In addition, we found that women who suffered IPV during their most recent pregnancy had a higher chance of having a preterm or low birthweight baby compared to those who did not experience any form of violence. IPV screening should be a regular part of ANC, so that pregnant women who are experiencing IPV can be monitored and supported to avoid adverse outcomes for their babies.

## Ethics and consent

Ethical clearance was obtained from the Ethics Review Committee of the Ghana Health Service, Accra, Ghana. Official permission was obtained from the Tamale Metropolitan Health Directorate. In addition, the study was conducted while taking into consideration the principles and ethics of health research. Participation was strictly voluntary, and written informed consent was obtained from respondents prior to administering the questionnaires. The nature of the study, including the objectives, risks, and potential benefits, was clearly explained to the respondents. Respondents were also informed about their right to withdraw at any time during the study. Confidentiality and anonymity were ensured throughout the execution of the study by excluding any information that could be linked to the respondents.

## Author contribution statement

Michael Boah; Abdul-Nasir Issah; Daudi Yeboah; Mary Rachael Kpordoxah; Jevaise Aballo: Conceived and designed the experiments; Performed the experiments; Analyzed and interpreted the data; Contributed reagents, materials, analysis tools or data; Wrote the paper.

Nashiru Abdulai; Martin Nyaaba Adokiya: Conceived and designed the experiments; Performed the experiments; Contributed reagents, materials, analysis tools or data; Wrote the paper.

## Funding

The study received no funding from any organization.

## Data availability statement

The data underlining the conclusions drawn in this study are contained within the manuscript. The dataset, however, can be made available on reasonable request from the corresponding author.

## Declaration of interest's statement

The authors declare that they have no known competing financial interests or personal relationships that could have appeared to influence the work reported in this paper.

## Abbreviations

ANC: Antenatal care
AOR: Adjusted odds ratio
APH: Antepartum Haemorrhage
CHPS: Community-based Health Planning and Services
CI: Confidence interval
DHS: Demographic and Health Survey
IPV: Intimate Partner Violence
IRB: Institutional Review Board
IUGR: Intrauterine Growth Restriction
LMICs: Low and Middle-Income Countries
SSA: sub-Saharan Africa
WHO: World Health Organization
