# Supplementary material for: Risk of adverse newborn outcomes among women who experienced physical and psychological intimate partner abuse during pregnancy in Ghana's northern region
Source: Heliyon. 2023 Apr 10;9(4):e15391. doi: 10.1016/j.heliyon.2023.e15391 (PMC10130875; doi:10.1016/j.heliyon.2023.e15391)
Supplement: Multimedia component 1 [file mmc1.docx]

Table 1: Percentage distribution of responses to questions on acts of physical and psychological intimate partner violence during recent pregnancy

| Physical violence | Frequency | Percentage |
| --- | --- | --- |
| Push you, shake, or throw something at you |  |  |
| No | 399 | 99.3 |
| Yes | 3 | 0.7 |
| Punch you with his fist or with something that could hurt you? |  |  |
| No | 393 | 97.8 |
| Yes | 9 | 2.2 |
| Kick you, drag you, or beat you up? |  |  |
| No | 398 | 99.0 |
| Yes | 4 | 1.0 |
| Try to choke you or burn you on purpose? |  |  |
| No | 402 | 100.0 |
| Yes | 0 | 0.0 |
| Threaten or attack you with a knife, gun or other weapon? |  |  |
| No | 402 | 100.0 |
| Yes | 0 | 0.0 |
| Twist your arm or pull your hair? |  |  |
| No | 382 | 95.0 |
| Yes | 20 | 5.0 |
| Psychological violence |  |  |
| Say or do something to humiliate you in front of others? |  |  |
| No | 388 | 96.6 |
| Yes | 14 | 3.4 |
| Threatened to hurt or harm you or someone you care about? |  |  |
| No | 399 | 99.3 |
| Yes | 3 | 0.7 |
| Insult or made you feel bad about yourself? |  |  |
| No | 264 | 65.9 |
| Yes | 138 | 34.1 |
